# Utilization of Electrochemical Sensors and Biosensors in Biochemistry and Molecular Biology

**DOI:** 10.3390/s8106125

**Published:** 2008-10-01

**Authors:** Vojtech Adam, Rene Kizek

**Affiliations:** 1 Department of Chemistry and Biochemistry Mendel University of Agriculture and Forestry, Zemedelska 1, CZ-613 00 Brno, Czech Republic; 2 Department of Animal Nutrition and Forage Production, Mendel University of Agriculture and Forestry, Zemedelska 1, CZ-613 00 Brno, Czech Republic

## Abstract

A special issue of *Sensors* entitled “Utilization of Electrochemical Sensors and Biosensors in Biochemistry and Molecular Biology” has been prepared over a period of three years. In this Editorial Note we would like to highlight one of the possible directions for electrochemical sensor and biosensor research resulting from the ideas of Czechoslovakian Nobel Prize winner Jaroslav Heyrovsky and his colleague Rudolf Brdicka.

## Could electrochemistry be employed in biochemistry and molecular biology in the 21^st^ century?

Cancer, “plague of the world”, is a word which brings to mind not only a threat to human health but also a subject of investigation for the broader scientific community (almost 60,000 papers appearing in Web of Science in 2007 included “cancer*” within their article titles, keywords or abstracts, and in particular, almost one hundred of them have been published in *Nature*). The success of treatment of the disease depends on many factors such as prevention, early and sensitive diagnostics. That means that the sooner a cancer is detected, the better the chances to treat it successfully. A very interesting idea about how we could diagnose a tumour disease was created seventy years ago in Czechoslovakia. This idea will not be forgotten.

Rudolf Brdicka, who was born one hundred years ago, published in *Nature* in 1937 his discoveries concerning the use of polarography to diagnose a tumour disease [1,2]. He discovered a sensitive polarographic “protein effect”, conspicuously exhibited by serum, which he explained as due to the catalytic activity of the sulphydryl groups of proteins. The “protein effect” consisted of the appearance of a characteristic wave on the current voltage curve, which was always found to be larger in normal serum samples than when the same procedure was applied to cancer serum [1,2]. A year ago Brdicka's colleague Jaroslav Heyrovsky, recipient of the 1959 Nobel Prize in Chemistry, published a paper in the same journal, where he summarized results obtained in the field of Polarographic Research on Cancer [3]. Heyrovsky believed that this field of study should be of general interest of many scientific groups around the world, but as it turns out he was apparently mistaken, as since then, electrochemistry has been slowly disappearing from tumour disease diagnostics due to the use of more modern analytical chemistry and molecular biology techniques. Thus, this unique and interesting technique has not been used, with several exceptions [4], for over fifty years.

## Metallothionein

Five years ago, we published in *Analytical Chemistry* a paper describing a highly sensitive determination of a protein – metallothionein [5]. This in itself would not be anything special, if we had not also revealed that this low molecular weight, intracellular, cysteine-rich protein has a certain connection with a tumour disease in the subsequent experiments.

## Is it a new possibility how we could diagnose a cancer?

As we have mentioned above, the sooner the cancer is detected, the better the chances to treat it successfully are. Thus, we used an electrochemical technique to analyse blood serum of patients with tumour diseases, the same way Brdicka did. Using a modified Brdicka reaction we have analyzed blood, blood serum and tumours of patients (more than 150) suffering from different types of cancers (melanoma, breast, lung, thyroid gland, kidney, oesophagus, large intestine, head and neck cancer, and leukaemia) and also blood and blood serum from non-cancer affected subjects. The results obtained show that the content of metallothionein increases markedly in blood and blood serum of the patients. We are the first scientific group in the world to not only quantify metallothionein in human blood serum, but also to report real differences between metallothionein levels of a control tissue and a tumourous one. Moreover, during thorough analysis of the experimental results obtained we found a unique correlation between the voltammogram data and the type of cancer. We called it an “electrochemical fingerprint”.

## Could electrochemistry really be utilized for cancer diagnostics in the 21^st^ century?

Could electrochemistry be used in 21^st^ century in addition to other robust techniques such as mass spectrometry, nuclear magnetic resonance and many others? One could raise the objection that this “old technique” cannot compete with these modern methods, but in practice the contrary is the case. The results obtained show that electrochemical techniques could be brought back for the investigation of cancer and could potentially enable easy, fast and low cost cancer diagnostics. It is thus possible that the wishes of Jaroslav Heyrovsky and his follower and colleague Rudolf Brdicka will be fulfilled and this auspicious field of cancer study will be developed intensively, not only in Czech laboratories but also around the world.

## Special issue “Utilization of Electrochemical Sensors and Biosensors in Biochemistry and Molecular Biology”

The special issue of *Sensors* contains more than thirty articles or reviews reporting on various fields of biological research. Experimental papers devoted to environmental stress at plants pay attention to the study of the influence of heavy metals on plant species and embryos. The most important feature of these papers was their multi-instrumental approach to find complex reactions of plants under stress [6-10]. Such a multi-instrumental approach was also employed in the paper reporting a non-destructive evaluation of historical papers [11].

Several papers report the use of miniaturized electrodes or miniaturized particles coupled with various detection techniques for the study of biochemical events and pathways [12-17]. As we mentioned at the beginning of this contribution, electrochemical techniques can be used in the diagnosis of tumour diseases [18, 19]. Moreover, these techniques can be also utilized for investigation of the interaction of anticancer drugs with various biologically active compounds, including glutathione, metallothionein and others [20, 21]. Attention was also paid to toxic substances such as bromadiolone, whch belongs to a group of second-generation anticoagulant rodenticides [22]. Other authors paid attention to the idea of using piezoelectric biosensors for a simple serological diagnosis of tularemia in hares [23]. Three papers were also published in which the authors used biochemical markers to study environmental pollution [24-26]. Similarly to these, new approaches and procedures to analyse cells and their behaviour were suggested [27].

Fruits and vegetables belong to the most important and crucial parts of human diet due to their health benefits. Plant tissues are rich in nutritionally or therapeutically active compounds, which are obviously products of plant secondary metabolism. Analysis of such compounds is therefore both important and needed. Moreover, new compounds belonging to the group of biologically active ones are continuosly being discovered. Sensor and biosensor research is also aimed at the developmenmt of easy-to-use and low cost detection of these substances [28-35].

Detection of nucleic acids is also of current interest, most of all due to recognition of their specific sequences. Approaches for studying nucleic acids, their interactions and modifications can differ, as shown in other papers published in the special issue [36-40].

## Figures and Tables

**Figure 1.**
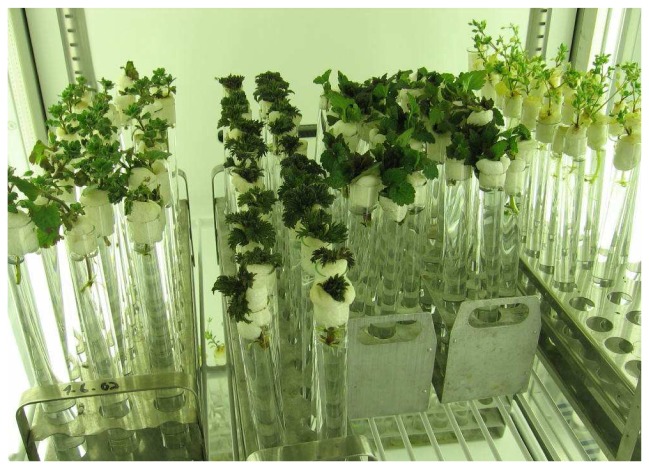
Picture of plants cultivated in an environmental chamber under controlled conditions.

**Figure 2.**
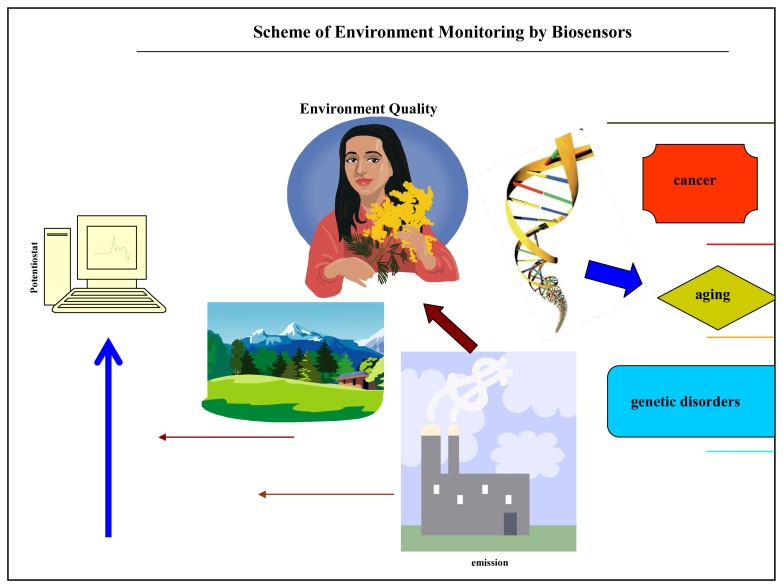
Schematic of environmental monitoring using biosensors.
